# Synthesis and Characterization of a Novel Hydroquinone Sulfonate-Based Redox Active Ionic Liquid

**DOI:** 10.3390/ma14123259

**Published:** 2021-06-12

**Authors:** Farida H. Aidoudi, Alessandro Sinopoli, Muthumeenal Arunachalam, Belabbes Merzougui, Brahim Aïssa

**Affiliations:** Qatar Environment and Energy Research Institute (QEERI), Hamad Bin Khalifa University (HBKU), Doha P.O. Box 34110, Qatar; aidoudifarida@gmail.com (F.H.A.); asinopoli@hbku.edu.qa (A.S.); msundarapandian@hbku.edu.qa (M.A.); belabbes613@gmail.com (B.M.)

**Keywords:** ionic liquid, redox-active ionic liquids, hydroquinone, cyclic voltammetry

## Abstract

Introducing redox-active moieties into an ionic liquid (IL) structure is an exciting and attractive approach that has received increasing interest over recent years for a various range of energy applications. The so-called redox-active ionic liquids (RAILs) provide a highly versatile platform to potentially create multifunctional electroactive materials. Ionic liquids are molten salts consisting of ionic species, often having a melting point lower than 100 °C. Such liquids are obtained by combining a bulky asymmetric organic cation and a small anion. Here, we report on the synthesis of a novel RAIL, namely 1-butyl-3-methylimidazolium hydroquinone sulfonate ((BMIM)(HQS)). (BMIM)(HQS) was synthesized in a two-step procedure, starting by the quaternization of methylimidazole using butylchloride to produce 1-butyl-3-methylimidazolium chloride ((BMIM)(Cl)), and followed by the anion exchange reaction, where the chloride anion is exchanged with hydroquinone sulfonate. The resulting product was characterized by ^1^H NMR, ^13^C NMR, FT-IR spectroscopy, themogravimetric analysis, and differential scanning calorimetry, and shows a high stability up to 340 °C. Its electrochemical behavior was investigated using cyclic voltammetry at different temperatures and its viscosity analysis was also performed at variable temperatures. The electrochemical response of the presented RAIL was found to be temperature dependent and diffusion controlled. Overall, our results demonstrated that (BMIM)(mix of HQS and HSQ) is redox active and possesses high stability and low volatility, leading to the employment of this RAIL without any additional supporting electrolyte or additives.

## 1. Introduction

Over the past two decades, ionic liquids (ILs) have received a great deal of attention, and extensive work has been devoted to developing their practical applications in various fields [1]. Many of the early studies have focused on the ‘green’ chemistry potential of these compounds, with a particular interest oriented towards replacing the organic solvents in homogeneous catalysis [2,3]. The unique property of ionic liquids that makes them environmentally suitable for these purposes is their intrinsic low vapor pressure, which has significant advantages when replacing highly volatile organic solvents [1]. Later on, ILs have been extensively studied in organic synthesis and materials chemistry [4,5], where they demonstrated their versatility and large range of applicability, not only as excellent solvents but, in many cases, in a more active role. For example, in the synthesis of porous materials (including zeolites and metal organic frameworks (MOFs)), ILs act as templates or structure directing agents, in addition to their principal role as reaction solvent [5]. Some physical and chemical key properties of ILs that make them attractive for many applications include their solubility and miscibility with water and many other solvents, intrinsic ionic conductivity, broad electrochemical potential window, good thermal stability, and very high boiling points [1].

However, the applicability of ILs is not limited only to the areas of catalysis and synthetic chemistry, as extensive research works have demonstrated their utility in diverse fields of technology, from energy applications, electropolishing, and electrodeposition to the use of supported ionic liquid as an electrocatalyst [6,7,8]. The application of ILs in the energy sector is likely the most appealing research area nowadays, especially for the development of energy conversion and storage materials and devices [9,10]. This is driven by the continuous need for the development of innovative systems in order to overcome the issues associated with the existing materials, and hence achieve the main goal of sustainable, safe, and cost-effective systems [9]. For example, lithium-ion batteries [11], despite their many advantages, still suffer from major safety issues, especially when they are employed on a large scale or high temperatures, due to the flammability of organic electrolytes. In this regard, ILs can serve as advanced materials to overcome these issues [12]. Another promising application could be represented by redox flow batteries (RFBs), which possess many attractive properties that make them excellent potential candidates for high energy density grid scale storage [13]. In this scenario, aqueous RFBs have limited cell voltage. While nonaqueous RFBs offer wider electrochemical windows, in exchange for the poor solubility of electroactive materials, which result in low energy density. In this context, the use of ILs for RFBs holds the potential to further advance this technology [14,15].

In addition to the many advantages of ILs previously described, the appeal of ILs in electrochemical applications is driven by their three key features, namely (i) their ionic conductivity, (ii) their large electrochemical potential window, and (iii) their thermal stability. The large electrochemical window may prevent side reactions, and the thermal stability allows for operation over wide ranges of temperatures. Another important feature of ILs that has attracted academic interest is the possibility of rationally altering their physical and chemical properties by varying the anion or the cation elements, or by introducing specific functionalities into the cation and/or anion, also known as task specific ILs (TSILs). This property stimulated the electrochemistry community to design novel ILs where the structure can be tailored to include redox active species; hence, they can be used not only as electrolytes but also as redox species or redox shuttles [16,17].

The concept of redox active IL (RAIL) is very intriguing, and research in this field is very promising, as this may pave the way for the development of new or improved electrochemical technologies [18,19].

ILs have widely been investigated as potential safe electrolytes for many electrochemical devices, and there are many reports on IL-based redox systems [20]. While some success was indeed achieved in terms of safety and increased cell voltages [21,22], the issue of solubility for these redox materials is still a major challenge [7]. In this context, RAILs present an elegant approach for increasing the concentration of redox active species in electrolytes, while overcoming the issue of poor solubility. This strategy extends the practicability of ILs beyond they mere role of inert electrolytes.

It is worth mentioning that ionic liquids with redox active moieties were know even before the modern definition of RAILs was introduced. This can be demonstrated by the work reported by Shreeve et al. [23], Kumar et al. [24] and Liu et al. [25] on the first ferrocenylmethylimidazolium and ferrocenylmethyltriazolium, the first viologen-based liquid crystalline containing “bis trifluromethylsulfonylimide” (TFSI)) as counter anion, and the first homogeneous mixture of vanadate, 2,2,6,6-tetramethyl-1-piperidinyloxy (TEMPO), acid functionalized ionic liquids, respectively. However, an enormous interest was devoted to the synthesis and application of these systems in energy in the past decade. This is driven by the need to achieve a liquid state system with high redox density. Some important recent studies on metal- and nonmetal-based RAILs are highlighted in Table 1, and a few selected examples are discussed below. Very recently, Sagara et al. [26] reported a charge transfer (CT) RAIL prepared by mixing carbazole-based and viologen-based RAILs. In their study, it was shown that mixing the two RAILs lead to unique properties that are distinct from those of the isolated components. Wang et al. [27] synthesized a RAIL bearing the redox active 2,2,6,6-tetramethyl-1-piperidinyloxy bis trifluromethylsulfonylimide (TFSI) for potential application in lithium oxygen batteries. It was demonstrated that adding this RAIL to diethylene glycol diethyl ether (DEGDME) leads to exceptional properties compared to pure DEGDME. A family of RAILs bearing the anthraquinone sulfonate (AQS) as anionic counterpart [28] were reported with different cations including phosphonium, ammonium, imidazolium, piperidinium, and pyrrolidinium. Among them only (P14,6,6,6) was found to be liquid at RT; this can be explained by the symmetry and planarity of the AQS core, coupled with the significant pi–pi intermolecular interaction between anthraquinone moieties, which favor a long-range order rather than liquefaction. In the attempt to achieve an ideal liquid system with a density of redox species close to the solid state, an excellent work has been reported by Fontaine et al. [29] on a bi-redox IL comprising a triflimide anion bearing the redox active anthraquinone (AQ–TFSI) and a methyl imidazolium cation bearing the redox active TEMPO. Rochford et al. [30] reported the synthesis of a RAIL based on 2,5-ditert-butyl-1,4-dimethoxybenzene (DDB) and a triflimide anion. This RAIL was highly soluble in carbonate-based electrolytes and holds the potential to be an excellent functional additive in Li-ion battery electrolytes, as it can act as redox shuttle for Li_4_Ti_5_O_12_/LiFePO_4_ lithium-ion batteries.

Despite the significant advancements in the field of RAILs, the development of an optimized system in the liquid state offering a high density of redox species still remains very challenging.

While functionalizing ILs and introducing electroactive species is relatively straightforward, and might be easily accomplished using the common preparative methods for the synthesis of TSILs, [31] developing RAILs add other requirements, which make their preparation very challenging. As a matter of fact, it is well known that a fairly narrow range of ILs structures are liquids at RT, and only a very few of the reported RAILs are liquids at RT [15,19,23,25,32,33], as illustrated in Table 1.

Hydroquinone is a simple typical and important electroactive molecule well-known in fundamental electrochemistry research [36]. Despite being heavily studied, there is no report, to the best of our knowledge, of using this simple molecule as a component of RAIL. Although, and as mentioned above, some RAILs having bulkier molecules (such as viologen [28] and anthraquinone [37]) have been reported, the vast majority of the electrochemical studies of these RAILs were reported using an additional solvent such as acetonitrile [37,38]. The simplicity of the hydroquinone anion combined with a suitable cation may result in a RAILs that is liquid at room temperature (RT) with an optimum viscosity. This property is crucial in order to achieve solvent free electrochemical systems for many energy storage applications (such as redox flow batteries).

**Figure 1:**
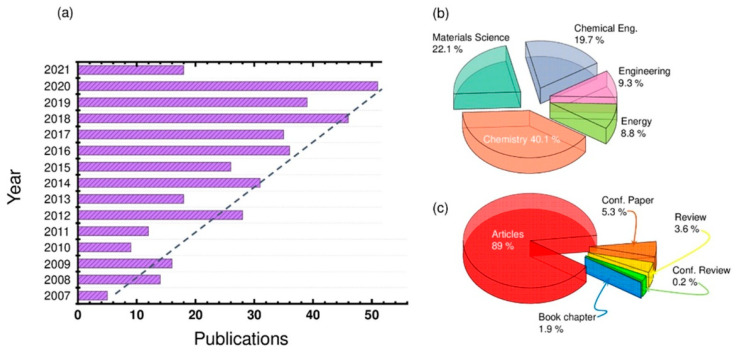
(**a**) Histogram distribution of the studies performed on the redox-active ionic liquids (RAILs) field, from 2007 to date, and (**b**) their respective distribution of the publications per discipline along with (**c**) the type of published documents. All published languages were included. All document types, including journal and conference articles, report paper, conference proceeding, and monograph published chapters were recorded. Statistics are available from 2007 to April 2021, inclusively. Data were collected from Scopus Expertly curated abstract and citation database based information service.

Recently, the topic of redox-active ionic liquids (RAILs) has resulted in a tremendous increase in terms of publications. Figure 1a displays the recent references related to the RAIL field from 2007 to 2021, showing a constant and continual increase. Looking to Figure 1b, one might notice that the most influential disciplines are Chemistry, Materials Science, and Chemical Engineering with emergence in other closely related fields such as Energy. While the scientific articles are dominating this publication domain, as shown in Figure 1c, only a small percentage of reviews articles are available so far (~3.6% of the published documents), and it would be interesting to contribute to filling this critical gap.

As part of our research interest in developing this burgeoning area further, we here report the synthesis and characterization of a new electrochemically active ionic liquid, 1-butyl-3-methyimidazolium hydroquinone sulfonate ((BMIM)(HQS)), having the redox species hydroquinone sulfonate as the anion. The studied RAIL result liquid at room temperature and characterized by a low water content. The electrochemical response of the developed RAIL was investigated at a wide range of temperatures, ranging from 20 to 70 °C, without any additional supporting electrolyte or additives. Viscosity and thermogravimetry analysis confirmed the high stability and low volatility of (BMIM)(HQS).

## 2. Materials and Methods

All chemicals were purchased from Sigma Aldrich (Saint Luis, MO, USA) and used without further purification. All reactions were carried out under inert nitrogen atmosphere. 

Products were characterized using ^1^H NMR, ^13^C NMR, FT-IR and TGA. Samples for NMR analyses were prepared by dissolving 20 mg of the product in about 0.5 mL of deutrated solvent (CDCl_3_/CD_3_OD). NMR spectra were recorded on a Bruker AVANCE III HD 800 MHz NMR spectrometer (Billerica, MA, USA) equipped with a 5 mm BBO H&F cryoprobe. NMR data were reported using the Aspin–NMR Data Reporting Tool (V 1.0). [39] FT-IR measurements were carried out using a Thermo Scientific Nicolet iS50 FTIR spectrometer (Waltham, MA, USA), via the KBr pellets method. The mass of the products was recorded using Thermo Scientific TSQ Quantitativa Quadrupole Mass Spectrometer (Waltham, MA, USA) by dissolving the samples in methanol.

The thermal stability of the products was examined by the thermogravimetric analyzer TA SDT Q600 TGA (New Castle, DE, USA). Samples were heated over temperatures between 20 to 600 °C under an oxygen atmosphere at a heating rate of 20 °C/min. In addition, melting point was measured with DSC-Q2000 (TA Instruments, New Castle, DE, USA) differential scanning calorimetry. The samples for DSC analysis were 10–20 mg; the heating and cooling rate was 10 °C min^−1^ over a temperature range of −70–+150 °C and the carrier gas was nitrogen. The samples were placed in an aluminum crucible. The DSC traces are reported in the ESI.

All electrochemical measurements were performed on SP-300 potentiostat controlled by EC-Lab software using a Three-electrode cell setup with a 5 mm diameter glassy carbon as working electrode, Ag/AgCl reference electrode and a platinum mesh counter electrode. Cyclic voltammetry was carried out using 10 mV/s scan rate and was performed at temperatures ranging from RT (21 °C) to 70 °C.

Viscosity was measured using Discovery HR-2 hybrid rheometer at temperatures ranging from 20 to 70 °C.

The water content of (BMIM)(HQS) was measured with a Metrohm 831KF titrator (Herisau, Switzerland), using HYDRANAL–Coulomat AG as an anolyte (Illkirch-Graffenstaden, France).

### Synthesis of (BMIM)(HQS)

**Scheme 1:**
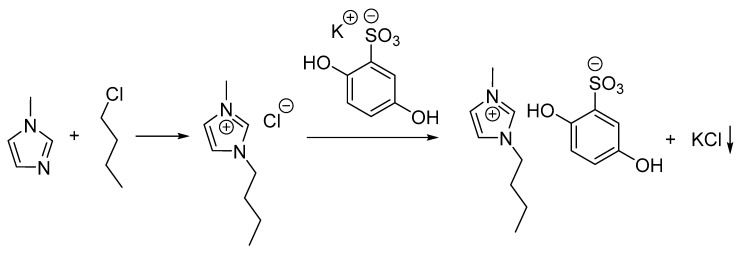
Synthetic pathway for the preparation of the redox-active ionic liquid.

The IL (BMIM)(HQS) was synthesized in a two-step procedure as illustrated in Scheme 1: (1) quaternization of methylimidazole using butylchloride to produce 1-butyl-3-methylimidazolium chloride ((BMIM)(Cl)); (2) anion exchange reaction was carried out in methanol using a slightly excess of potassium hydroquinone sulfonate (1.15 eq.).

The exact procedure is described below:

*(i)*  *Synthesis of (BMIM)(Cl)*

Under inert atmosphere conditions, degassed butylchloride (77.64 g, 0.566 mol) was added to redistilled N-methylimidazole (39.64 g, 0.482 mol) with constant stirring. The mixture was refluxed at 70 °C for 72 h under nitrogen atmosphere. After completion, the reaction mixture was then cooled to room temperature. Ethyl acetate was added, and the product was crystallized from the solution. It was filtered, washed with ethyl acetate, and dried under vacuum for 24 h yielding a white solid. Yield: 94%.

The product was stored under an inert atmosphere. ^1^H NMR (800 MHz, CDCl_3_) δ (ppm): 10.55 (s, 1H, NCHN); 7.65 (s, 1H, CH_3_NCHCHN); 7.48 (s, 1H CH_3_NCHCHN); 4.27 (*t*, *J* = 7.43 Hz, 2H, NCH_2_(CH_2_)_2_CH_3_); 4.06 (s, 3H, NCH_3_); 1.83 (pent, *J* = 7.58 Hz, 2H, NCH_2_CH_2_CH_2_CH_3_); 1.31 (sext, *J* = 7.56 Hz, 2H, N(CH_2_)_2_CH_2_CH_3_); 0.89 (*t*, *J* = 7.50 Hz, 3H, N(CH_2_)_3_CH_3_). ^13^C NMR (200 MHz, CDCl_3_) δ (ppm): 137.79, 123.71, 122.01, 49.71, 36.48, 32.15, 19.43, 13.42. IR: 3138 cm^−1^–2735 cm^−1^ (aromatic and aliphatic C–H), 1645 cm^−1^ (C=C-), 1168 cm^−1^ (C=N), and 1570 cm^−1^, (C–N). Melting point: +53 °C. Mass spectrum (Quad +ve) *m*/*z*: 313.33 (((BMIM)_2_Cl^−^}), 138.6 ((BMIM)^+^).

*(ii)*  *Synthesis of (BMIM)(HQS)*

(BMIM)(Cl) (24 g, 0.137 mol) was dissolved in 40 mL of methanol. After that, an excess of potassium hydroquinone sulfonate (36 g, 0.157 mol) was added. The mixture was stirred at room temperature (21 °C) for 72 h, after which the KCl precipitate was removed by filtration. Then dry acetone was added, and the precipitate was removed via filtration. This last step was repeated 3–4 times until no further precipitation was observed. Solvents were removed using rotavapor and the resulting product was dried under high vacuum at 60 °C for 24 h. Yield: 70%.

The viscous liquid product was stored under inert atmosphere. ^1^H NMR (800 MHz, CD_3_OD) δ (ppm): 8.89 (s, attenuated); 7.59 (s, 1H, CH_3_NCHCHN); 7.52 (s, 1H, CH_3_NCHCHN); 7.06 (d, *J* = 1.76 Hz, 1H, SCCHCO); 6.75 (dd, *J* = 8.76, 1.92 Hz, 1H, SCCHCOCH); 6.7 (d, *J* = 8.80 Hz, 1H, SCCOCH); 4.18 (*t*, *J* = 7.42 Hz, 2H, NCH_2_(CH_2_)_2_CH_3_); 3.90 (s, 3H, NCH_3_); 1.84 (pent, *J* = 7.49 Hz, 2H, NCH_2_CH_2_CH_2_CH_3_); 1.35 (sext, *J* = 7.52 Hz, 2H, N(CH_2_)_2_CH_2_CH_3_); 0.97 (*t*, *J* = 7.48 Hz, 3H, N(CH_2_)_3_CH_3_). ^13^C NMR (200 MHz, CD_3_OD) δ (ppm): 150.8, 147.96, 137.84, 129.86, 124.92, 123.63, 120.76, 118.82, 114.1, 50.58, 36.46, 33.09, 20.44, 13.78. IR: 3150 cm^−1^–2870 cm^−1^ (aromatic and aliphatic C–H), 1610 cm^−1^ (C=C), 1162 cm^−1^ (C=N), and 1570 cm^−1^ (C–N), 1230 cm^−1^ (S=O), 1072 cm^−1^ (S–O). Melting point: -41 °C. Mass spectrum (Quad +ve) *m*/*z*: 138.89 ((BMIM)^+^), −ve) *m*/*z*: 189.8 ((HSQ)^−^).

## 3. Results and Discussion

^1^H NMR and ^13^C NMR confirm the molecular structure of (BMIM)(Cl), and NMR data are comparable with the literature [40] (NMR spectra are reported in the ESI). The successful exchange of the chloride with hydroquinone sulfonate in (BMIM)(HQS) is clearly indicated in both ^1^H NMR and ^13^C NMR by the presence of the characteristic peaks of hydroquinone sulfonate with three additional peaks, observed in ^1^H NMR of (BMIM)(HQS) in the range between 6.6–7.2 ppm, attributed to the aromatic protons in HQS. In addition to that, ^13^C NMR spectrum illustrates the characteristic additional carbon peaks of hydroquinone sulfonate in the 110–160 ppm range. These results clearly demonstrate that the synthesis of (BMIM)(HQS) was successfully achieved. The ^1^H NMR analysis for (BMIM)(HQS) revealed a strong attenuation of the NCHN peak (BMIM fragment) speculatively attributed to the acidity of this proton and accordingly a proton exchange with the HQS fragment or with the solvent.

**Figure 2:**
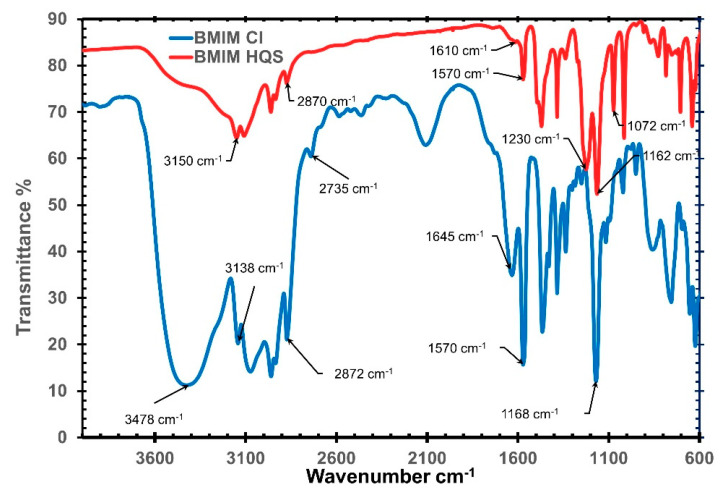
Infrared spectra for (BMIM)(Cl) and (BMIM)(HQS).

The incorporation of hydroquinone sulfonate as an anion in (BMIM)(HQS) was further confirmed using FT-IR. In the FT-IR, when infrared photons hit the sample, some wavelengths are absorbed while other pass through (i.e., transmitted). The resulting signal spectrum represent a footprint of the sample, with typical fingerprint signal for specific molecular functional groups. As shown in Figure 2, the IR spectrum for (BMIM)(HQS) shows the clear characteristic (S=O) peak located in the range of 1322 −1149 cm^−1^ and (S–O) peak at around 1072 cm^−1^. In addition to that, the large broad peak in the range of 3400–3300 cm^−1^ in (BMIM)(Cl) is not present in (BMIM)(HQS). The presence of this specific peak usually refers to an extensive H-bonding network between water molecules and the imidazolium cations [41]. As chloride ions are silent towards NMR analysis, and the FT-IR spectra of the ILs here reported do not provide quantitative information, to estimate the purity of (BMIM)(HQS) we have performed gravimetric silver nitrate test. This revealed the presence of 3.8% mol/mol of chloride traces with respect to (BMIM)(HQS).

Coulometric water content determination is primarily used for the determination of small amounts of water in the ppm range. In a coulometric titrator, electric current is used to generate the iodine needed for the Karl Fischer reaction. The current releases a stoichiometrically corresponding amount of iodine from the iodide-containing reagent. (BMIM)(Cl) is found to be highly hygroscopic and is more prone to quick hydration by absorbing moisture from the air. In contrast, (BMIM)(HQS) is more stable in air and does not absorb moisture, despite being soluble in water. This has been confirmed by the coulometric KF titration method. The moisture content in (BMIM)(HQS) was found to be 0.1% whereas (BMIM)(Cl) has been reported around 6.85% [42]. This is a relevant and representative example that demonstrates how changing the ILs anion can efficiently affect the final physical properties of the compound.

**Figure 3:**
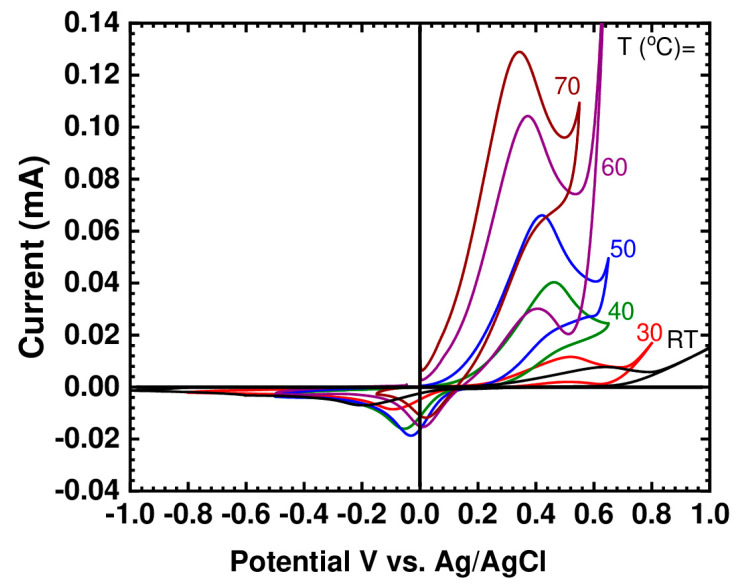
CV of the pure (BMIM)(HQS) at selected temperatures using glassy carbon working electrode, at 10 mV/s scan rate.

Figure 3 shows the cyclic voltammogram for (BMIM)(HQS) recorded at different temperatures. At room temperature (21 °C) the potential difference between the anodic peak and its associated cathodic peak (ΔE = 0.8 V) is high enough to indicate that the kinetic is fairly slow. Similar behavior was previously reported for the electrochemistry of hydroquinone dissolved in the non-active IL 1-butyl-3-methyimidazolium hexafluorophosphate (BMIM)(PF_6_) and 1-butyl-3-methylimidazolium tetrafluroborate (BMIM)(BF_4_) [37]. The same trend was also reported for the behavior of hydroquinone in acetonitrile [38]. Instead, reduction processes at the BMIM cationic fragment typically occur at more negative potentials, outside the electrochemical window here investigated [43].

**Figure 4:**
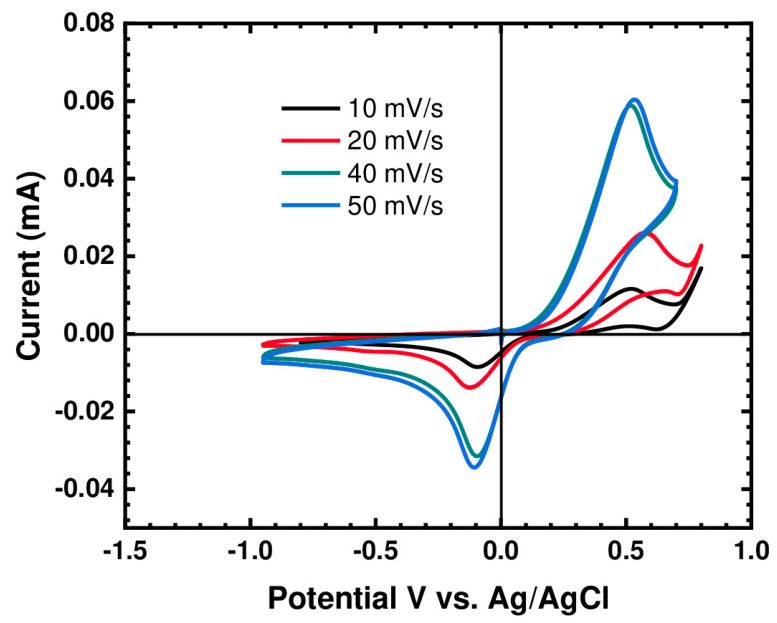
CV of (BMIM)(HQS) at 30 °C using glassy carbon working electrode, at various scan rates.

**Figure 5:**
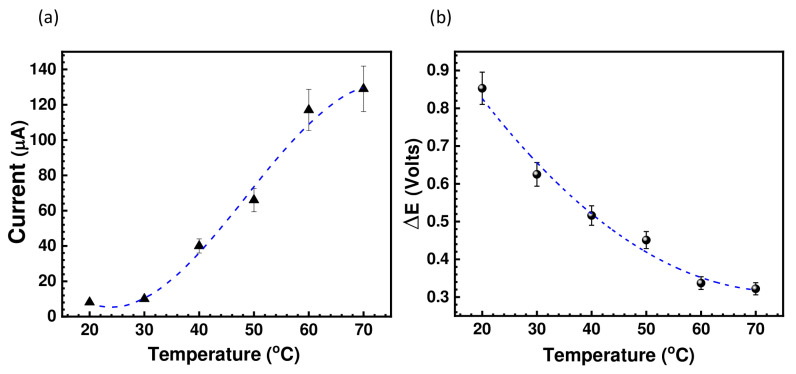
Variation of (**a**) the peak current as a function of the temperature, and (**b**) variation of redox potential as function of the temperature (right) for (BMIM)(HQS).

The cyclic voltammograms depicted in Figure 4 show a clear dependence of the peak positions and peak current intensity on the temperature. It is clear that there is a significant reduction in peak potential separation (ΔE); hence, the kinetics were improved at higher temperatures, together with a significant increase in the peak current intensity as shown in Figure 5. This indicates that increasing the temperature considerably reduces the viscosity of the IL, which allows for an increase in the diffusion rate of the active species.

In general, when ΔE is higher than 600 mV, the hydroquinone/quinone system is considered quasireversible or slow. However, in this system, the active species (HQ/Q) are in the form of ionic liquid and, as explained above, the viscosity can greatly affect the cyclic voltammetry behavior. This can be demonstrated from the electrode response upon increased temperature. As temperature increases, ΔE decreases. This indicates that the system is reversible, but its electrochemical behavior is being affected by the viscosity. Furthermore, the HQ/Q redox couple is well known for its reversibility; nevertheless, such reversibility (the degree of the anodic and cathodic peak) strongly depends on the nature of the working electrode and electrolyte (medium). Figure 4 shows the cyclic voltammograms of our system using various scan rate at 30 °C. It is evident that the peak current increases nonlinearly with an increasing the scan rate, suggesting a diffusion driven electrochemical redox process. This is not surprising, and a similar electrochemical behavior was reported on a family of metal-based ionic liquids [15].

**Figure 6:**
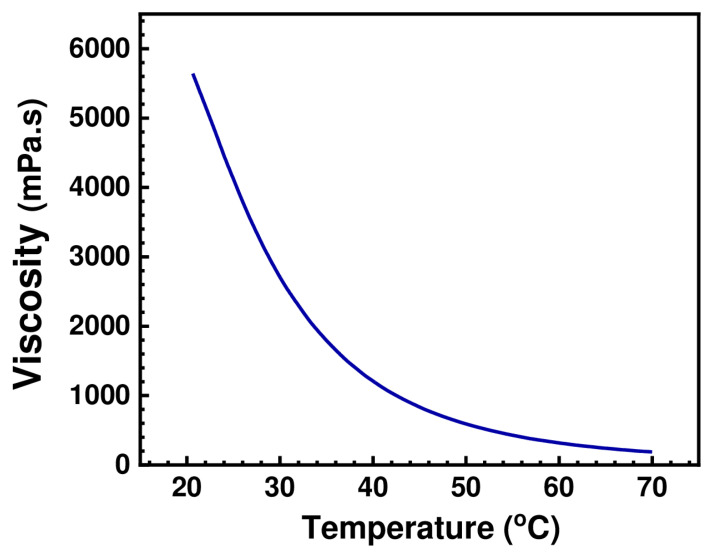
The viscosity of (BMIM)(HQS) in the 20–70 °C temperature range.

As illustrated in Figure 6, the effect of increasing the temperature on the viscosity of (BMIM)(HSQ) was experimentally validated by measuring the viscosity of (BMIM)(HSQ) over the temperatures range 20–70 °C. The initial viscosity of 5640 mPa·s drastically dropped with increasing the temperature, reaching 186 mPa·s at 70 °C. This is a crucial factor to consider, since a redox flow battery is likely to be operated at different temperatures. The measured viscosities are anyway higher than those reported for imidazole-based ILs, which usually do not exceed the value of 160 mPa·s at 10 °C [42,44]. On the other hand, the high viscosity of (BMIM)(HQS) is counterbalanced by its low volatility and high thermal stability as discussed below.

**Figure 7:**
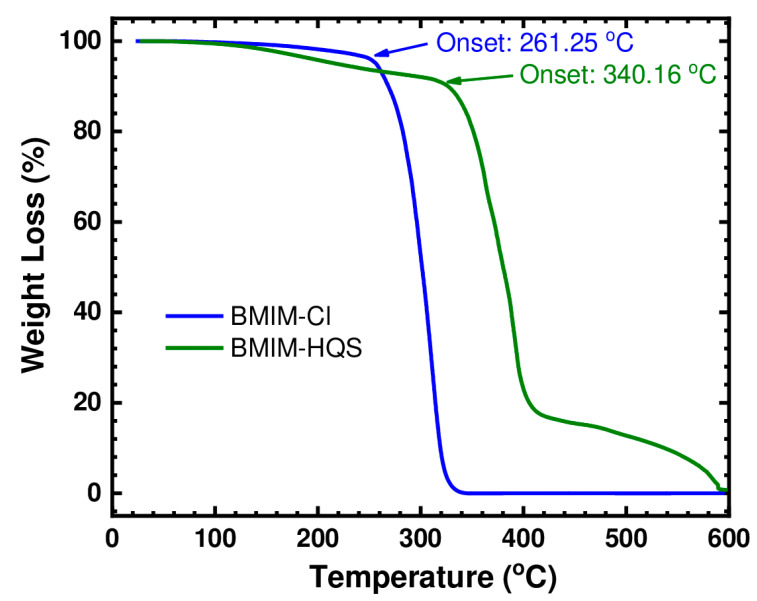
TGA analysis of (BMIM)(Cl) and (BMIM)(HSQ).

The thermal stability of (BMIM)(Cl) and (BMIM)(HQS) was determined by thermo-gravimetric analyzer in order to know their upper temperature limit. (BMIM)(HQS) resulted in a better thermal stability than (BMIM)(Cl), based on the TGA graph in Figure 7. The onset of thermal decomposition started at 261 °C for (BMIM)(Cl), as previously reported in the literature [45], whereas (BMIM)(HQS) started decomposing around 340 °C. This shows a high thermal stability for the RAIL here reported. It well established that anion type plays a major role in determining thermal stability, similar to determining the hydrophobicity of ILs [46,47]. Previous investigations have reported that the thermal stability of ILs with different anions decreases with the increase of the coordinating nature, nucleophilicity, and hydrophilicity of the corresponding anion [46]. This is in agreement with the difference of 80 °C in the onset temperature here measured in favor of HQS anion with respect to Cl^−^. Indeed, it is well known that ILs properties can be tuned by varying the cation or the anion or both. This work and several other previous theoretical and experimental studies [48,49,50,51,52,53] clearly demonstrated that changing the ionic liquid anion greatly affects its physical properties (including viscosity, thermal stability, and melting point). As a matter of fact, in a study on several ionic liquids containing macrocyclic fragments [49], it was clearly demonstrated that replacing the halide anions by TFSI anions considerably decrease the melting point of the resulting IL. Similarly in another study [48], the ionic liquid 1-butyl-3-methylimidazolium bis(trifluoromethyl) phosphinate (BMIM (CF_3_)2PO_2_) did not show a melting point in the temperature range −120 to 100 °C; in contrast, BMIM TFSI has a melting point of −3 °C [48].

## 4. Conclusions

The use of RTIL is supported by several crucial factors, which include low volatility, high concentration of redox-active species, high energy density, electrochemical and thermal stability so they can be used at moderate and even high temperatures. Here we reported the successful synthesis of (BMIM)(HSQ) bearing the electrochemically active hydroquinone sulfonate as an anion. The electrochemical characterization was performed on the sole RAIL with no other additives or solvents, revealing a diffusion driven electrochemical redox process, affected by a fairly slow kinetic. Replacement of the halide (chloride) anion with an organic (HSQ) anion resulted, as expected, in a lower water content of the final IL (higher hydrophobicity), together with a higher viscosity and higher thermal stability. This study demonstrates that ILs can be functionalized to include simple redox active species; thus, the RAILs can be used without any additional supporting electrolyte or additives. Of course, further studies are needed on the application of our system into electrochemical devices. For instance, studying the behavior of this novel RAIL with different electrode materials, including both carbonaceous and noncarbonaceous, will result in very useful findings. In this regard, we have already conducted an initial study using carbon paper as working electrode (JNTG carbon paper), which gave similar results compared to glassy carbon and the preliminary voltammograms are reported in the ESI.

In the future, the use of molecular engineering will add a further step to this study by modifying the anion/cation in order to achieve an optimum system with comparable stability but lower viscosity. Clearly, the flexibility and the possibility of designing a large number of hydroquinone-containing RTILs offer great opportunities for the development of new or improved electrochemical technologies, and the field is still widely open for further explorations and studies.

## Figures and Tables

**Table 1:** Example of selected RAILs, their representative cations and anions, physical state and potential applications.

RAIL	Cation	Anion	Physical State	Electrochemical Studies	Potential Application	Ref
Carbazole- and viologen based RAIL	(CZC_4_ImC_1_)^+^ + (C4VC7)^2+^	TFSI	Liquid at RT	Neat RAIL	Charge transfer (CT) in order to produce new photoactivity	[26]
TEMPO-based RAIL	1,2-dimethyl-3-(4-(2,2,6,6-tetramethyl-1-oxyl-4-piperidoxyl)-pentyl)imidazolium	TFSI	Viscous liquid	RAIL in DEGDME	Lithium–oxygen batteries	[27]
Methoxycarbonyl-based RAIL	1-ethyl4-(methylcarbonyl)pyridinium	TFSI	Viscous liquid at RT	RAIL in DMF containing 0.1 M TBAPF_6_	Visible light-driven B12 catalytic reactions	[34]
Ferrocenyl sulfonium-based RAIL	dimethylferrocenylsulfonium	TFSI	Viscous liquid	RAIL in EMIM TFSI	N/A	[33]
Ferrocene- and viologen-based RAILs composite	FcC_6_IMC_1_ + C_4_VC_7_	TFSI	Viscous liquid at RT	RAIL in acetonitrile containing 0.1 M potassium TFSI	Electrochromism	[19]
Iron-based RAIL	Fe(OHCH_2_CH_2_)_2_NH)_6_	CF_3_SO_3_	Viscous liquid at RT	0.0111 M solution of RAIL in BMIm-PF6	Nonaqueous RFBs	[35]
	Fe(EA)_6−m_(FcEA)_m_	CF_3_SO_3_/I	Solid or Viscous liquid at RT *	RAIL in PC with 0.5 M Li TFSI	Nonaqueous RFBs	[15]
Anthraquinone-based RAIL	Phosphonium—Imidazolium Ammonium—Piperidinium pyrrolidinium	9,10-Anthraquinone-2-sulfonate (AQS)	Only P14 6 6 6 Was liquid at RT	RAIL in acetonitrile and TBA TFB electrolyte	N/A	[28]
Dimethoxybenzene-based RAIL	BMIM	2,5-ditert-butyl-1,4-dimethoxybenzene TFSI	Solid at RT	RAIL in carbonated based electrolyte	Li-ion batteries	[30]
Anthraquinone and TEMPO-based RAIL	EMIM TEMPO	AQ-TFSI	Solid at RT	RAIL in acetonitrile containing 0.1 M TBAPF_6_	supercapacitors	[29]
Ferrocene-	Ferrocene	TSFI	Solid at RT	N/A	N/A	[23]
Viologen-based RAIL	Viologen	TSFI	Solid at RT	N/A	Electrochromism	[24]
Vanadate	N-dodecylpyridinium	vanadate	Solid at RT	N/A	Alcohol oxidation by H_2_O_2_	[25]

CZ: carbazole, Im: imidazolium, V: viologen, TFSI: bis(trifluoromethylsulfonylimide), TEMPO: tetramethyl-1-piperidinyloxy, DEGDME: diethylene glycol diethyl ether, TBA TFB: tetrabutylammonium tetrafluoroborate, BMIM: 1-butyl-3-methylimidazolium, AQ: anthraquinone, TBA PF6: tetrabutylammonium hexafluorophosphate, Fc: ferrocene, BMIM: 1-butyl-3-methylimidazolium. * The physical properties of RAILs depend on the degree of substitution with I and Fc. Cells shaded in grey represent early examples of RAILs.

## Data Availability

Not applicable.

## Supplementary Materials

The following are available online at https://www.mdpi.com/article/10.3390/ma14123259/s1, Figure S1: ^1^H NMR for (BMIM)(Cl), Figure S2: ^13^C NMR for (BMIM)(Cl), Figure S3: ^1^H NMR for (BMIM)(HQS), Figure S4: ^13^C NMR for (BMIM)(HQS), Figure S5: CV of the pure (BMIM)(HQS) over the temperature range RT—70 °C. The scan rate is 10 mV/s, the working electrode is a 300 µm thickness JNTG carbon paper, Figure S6: MS spectrum of (BMIM)(Cl) in positive mode, Figure S7: MS spectrum of (BMIM)(HQS) in positive mode, Figure S8: MS spectrum of (BMIM)(HQS) in negative mode.

## References

[1] MacFarlane D.R., Kar M., Pringle J.M. (2017). An Introduction to Ionic Liquids. Fundamentals of Ionic Liquids.

[2] Cole-Hamilton D.J. (2003). Homogeneous catalysis—New approaches to catalyst separation, recovery, and recycling. Science.

[3] Rogers R.D., Seddon K.R. (2003). Ionic liquids—Solvents of the future?. Science.

[4] Aidoudi F.H., Aldous D.W., Goff R.J., Slawin A.M.Z., Attfield J.P., Morris R.E., Lightfoot P. (2011). An ionothermally prepared S = 1/2 vanadium oxyfluoride kagome lattice. Nat. Chem..

[5] Morris R.E. (2009). Ionothermal synthesis—Ionic liquids as functional solvents in the preparation of crystalline materials. Chem. Commun..

[6] Watanabe M., Thomas M.L., Zhang S., Ueno K., Yasuda T., Dokko K. (2017). Application of ionic liquids to energy storage and conversion materials and devices. Chem. Rev..

[7] Salanne M., Kirchner B., Perlt E. (2018). Ionic Liquids for Supercapacitor Applications. Ionic Liquids II.

[8] Yu L., Chen G.Z. (2019). Ionic liquid-based electrolytes for supercapacitor and supercapattery. Front. Chem..

[9] Mishra A., Mehta A., Basu S., Malode S.J., Shetti N.P., Shukla S.S., Nadagouda M.N., Aminabhavi T.M. (2018). Electrode materials for lithium-ion batteries. Mater. Sci. Energy Technol..

[10] Yang Q., Li L., Lin C.X., Gao X.L., Zhao C.H., Zhang Q.G., Zhu A.M., Liu Q.L. (2018). Hyperbranched poly(arylene ether ketone) anion exchange membranes for fuel cells. J. Membr. Sci..

[11] Mishra A., Shetti N.P., Basu S., Reddy K.R., Aminabhavi T.M., Asiri A.M.I., Kanchi S. (2020). Chapter 7—Recent Developments in Ionic Liquid-Based Electrolytes for Energy Storage Supercapacitors and Rechargeable Batteries. Green Sustainable Process for Chemical and Environmental Engineering and Science.

[12] Yang Q., Zhang Z., Sun X.-G., Hu Y.-S., Xing H., Dai S. (2018). Ionic liquids and derived materials for lithium and sodium batteries. Chem. Soc. Rev..

[13] Darling R.M., Gallagher K.G., Kowalski J.A., Ha S., Brushett F.R. (2014). Pathways to low-cost electrochemical energy storage: A comparison of aqueous and nonaqueous flow batteries. Energy Environ. Sci..

[14] Anderson T.M., Pratt H. (2015). Ionic Liquid Flow Battery Materials and Prototyping (No. SAND2015-2099C).

[15] Small L.J., Pratt H.D., Staiger C.L., Anderson T.M. (2017). MetILs3: A strategy for high density energy storage using redox-active ionic liquids. Adv. Sustain. Syst..

[16] Chakrabarti M.H., Mjalli F.S., AlNashef I.M., Hashim M.A., Hussain M.A., Bahadori L., Low C.T.J. (2014). Prospects of applying ionic liquids and deep eutectic solvents for renewable energy storage by means of redox flow batteries. Ren. Sustain. Energy Rev..

[17] Winsberg J., Hagemann T., Janoschka T., Hager M.D., Schubert U.S. (2017). Redox-flow batteries: From metals to organic redox-active materials. Angew. Chem. Int. Ed..

[18] Rochefort D. (2019). Enabling new electrochemical methods with redox-active ionic liquids. Curr. Opin. Electrochem..

[19] Tahara H., Uranaka K., Hirano M., Ikeda T., Sagara T., Murakami H. (2019). Electrochromism of ferrocene- and viologen-based redox-active ionic liquids composite. ACS Appl. Mater. Interfaces.

[20] Giffin G.A. (2016). Ionic liquid-based electrolytes for “beyond lithium” battery technologies. J. Mater. Chem. A.

[21] Liu K., Wang Z., Shi L., Jungsuttiwong S., Yuan S. (2021). Ionic liquids for high performance lithium metal batteries. J. Energy Chem..

[22] Ohno H. (2017). Electrochemistry of and in Ionic Liquids. Fundamentals of Ionic Liquids.

[23] Gao Y., Twamley B., Shreeve J.N.M. (2004). The first (ferrocenylmethyl)imidazolium and (ferrocenylmethyl)triazolium room temperature ionic liquids. Inorg. Chem..

[24] Bhowmik P.K., Han H., Cebe J.J., Burchett R.A., Acharya B., Kumar S. (2003). Ambient temperature thermotropic liquid crystalline viologen bis(triflimide) salts. Liq. Cryst..

[25] Wang S.-S., Popović Z., Wu H.-H., Liu Y. (2011). A homogeneous mixture composed of vanadate, acid, and TEMPO functionalized ionic liquids for alcohol oxidation by H_2_O_2_. ChemCatChem.

[26] Tahara H., Tanaka Y., Yamamoto S., Yonemori S., Chan B., Murakami H., Sagara T. (2021). A redox-active ionic liquid manifesting charge-transfer interaction between a viologen and carbazole and its effect on the viscosity, ionic conductivity, and redox process of the viologen. Chem. Sci..

[27] Zhang J., Sun B., Zhao Y., Tkacheva A., Liu Z., Yan K., Guo X., McDonagh A.M., Shanmukaraj D., Wang C. (2019). A versatile functionalized ionic liquid to boost the solution-mediated performances of lithium-oxygen batteries. Nat. Commun..

[28] Doherty A., Patterson S., Diaconu L., Graham L., Barhdadi R., Puchelle V., Wagner K., Office D., Chen J., Wallace G. (2015). Quinone redox-active ionic liquids. J. Mex. Chem. Soc..

[29] Mourad E., Coustan L., Lannelongue P., Zigah D., Mehdi A., Vioux A., Freunberger S.A., Favier F., Fontaine O. (2017). Biredox ionic liquids with solid-like redox density in the liquid state for high-energy supercapacitors. Nat. Mater..

[30] Gélinas B., Bibienne T., Dollé M., Rochefort D. (2017). Electroactive ionic liquids based on 2,5-ditert-butyl-1,4-dimethoxybenzene and triflimide anion as redox shuttle for Li_4_Ti_5_O_12_/LiFePO_4_ lithium-ion batteries. J. Power Sources.

[31] Sawant A.D., Raut D.G., Darvatkar N.B., Salunkhe M.M. (2011). Recent developments of task-specific ionic liquids in organic synthesis. Green Chem. Lett. Rev..

[32] Aldous L., Black J.J., Elias M.C., Gélinas B., Rochefort D. (2017). Enhancing thermoelectrochemical properties by tethering ferrocene to the anion or cation of ionic liquids: Altered thermodynamics and solubility. Phys. Chem. Chem. Phys..

[33] Venker A., Vollgraff T., Sundermeyer J. (2018). Ferrocenyl-sulfonium ionic liquids—Synthesis, characterization and electrochemistry. Dalton Trans..

[34] Shimakoshi H., Houfuku N., Chen L., Hisaeda Y. (2019). Redox active ionic liquid as efficient mediator and solvent for visible light-driven B12 catalytic reactions. Green Energy Environ..

[35] Anderson T.M., Ingersoll D., Rose A.J., Staiger C.L., Leonard J.C. (2010). Synthesis of an ionic liquid with an iron coordination cation. Dalton Trans..

[36] Guo Y., He D., Xie A., Qu W., Tang Y., Zhou L., Zhu R. (2019). The electrochemical oxidation of hydroquinone and catechol through a novel poly-geminal dicationic ionic liquid (PGDIL)–TiO_2_ composite film electrode. Polymers.

[37] Bhat M. (2012). Mechanistic, kinetic and electroanalytical aspects of quinone–hydroquinone redox system in N-alkylimidazolium based room temperature ionic liquids. Electrochim. Acta.

[38] Salazar R., Vidal J., Martínez-Cifuentes M., Araya-Maturana R., Ramírez-Rodríguez O. (2015). Electrochemical characterization of hydroquinone derivatives with different substituents in acetonitrile. New J. Chem..

[39] Sinopoli F., Sinopoli A. (2019). ASpin-NMR data reporting tool. Open J. Chem..

[40] Shekaari H., Zafarani-Moattar M.T., Mirheydari S.N. (2016). Effect of 1-butyl-3-methylimidazolium ibuprofenate as an active pharmaceutical ingredient ionic liquid (API-IL) on the thermodynamic properties of glycine and L-alanine in aqueous solutions at different temperatures. J. Solut. Chem..

[41] Voss J.M., Marsh B.M., Zhou J., Garand E. (2016). Interaction between ionic liquid cation and water: Infrared predissociation study of [bmim] +·(H_2_O)n clusters. Phys. Chem. Chem. Phys..

[42] Dharaskar S.A., Varma M.N., Shende D.Z., Yoo C.K., Wasewar K.L. (2013). Synthesis, characterization and application of 1-butyl-3 methylimidazolium chloride as green material for extractive desulfurization of liquid fuel. Sci. World J..

[43] Shamsipur M., Beigi A.A.M., Teymouri M., Pourmortazavi S.M., Irandoust M. (2010). Physical and electrochemical properties of ionic liquids 1-ethyl-3-methylimidazolium tetrafluoroborate, 1-butyl-3-methylimidazolium trifluoromethanesulfonate and 1-butyl-1-methylpyrrolidinium bis(trifluoromethylsulfonyl)imide. J. Mol. Liq..

[44] Barthen P., Frank W., Ignatiev N. (2015). Development of low viscous ionic liquids: The dependence of the viscosity on the mass of the ions. Ionics.

[45] Efimova A., Hubrig G., Schmidt P. (2013). Thermal stability and crystallization behavior of imidazolium halide ionic liquids. Thermochim. Acta.

[46] Cao Y., Mu T. (2014). Comprehensive Investigation on the Thermal Stability of 66 Ionic Liquids by Thermogravimetric Analysis. Ind. Eng. Chem. Res..

[47] Aïssa B., Hamoudi Z., Takahashi H., Tohji K., Mohamedi M., Khakani M.A.E. (2009). Carbon nanohorns-coated microfibers for use as free-standing electrodes for electrochemical power sources. Electrochem. Commun..

[48] Herath M.B., Hickman T., Creager S.E., DesMarteau D.D. (2011). A new fluorinated anion for room-temperature ionic liquids. J. Fluor. Chem..

[49] Pavel L.P., Andreyko E.A., Gorbatova P.A., Parfenov V.V., Rizvanov I.K., Stoikov I.I. (2017). Towards macrocyclic ionic liquids: Novel ammonium salts based on tetrasubstituted p-tert-butylthiacalix[4]arenes. RSC Adv..

[50] Sánchez-Ramírez N., Assresahegn B.D., Bélanger D., Torresi R.M. (2017). A comparison among viscosity, density, conductivity, and electrochemical windows of N-n-butyl-N-methylpyrrolidinium and triethyl-n-pentylphosphonium bis(fluorosulfonyl imide) ionic liquids and their analogues containing bis(trifluoromethylsulfonyl) imide anion. J. Chem. Eng. Data.

[51] Roohi H., Iloukhani H., Rouhani F. (2017). Tuning the structural, electronic and electrochemical properties of the 4-methyl-1-phenyl triazolium based [PhMeTAZ][Y1–8] ionic liquids through changing anions: A quantum chemical study. J. Mol. Liq..

[52] McDaniel J.G., Son C.Y., Yethiraj A. (2018). Ab initio force fields for organic Anions: Properties of (BMIM)[TFSI], (BMIM)[FSI], and (BMIM)[OTf] ionic liquids. J. Phys. Chem. B.

[53] Dai J., Zhao K.-Q., Wang B.-Q., Hu P., Heinrich B., Donnio B. (2020). Liquid crystal ionic self-assembly and anion-selective photoluminescence in discotic azatriphenylenes. J. Mater. Chem. C.

