# Supplementary material for: Synthesis and Characterization of a Novel Hydroquinone Sulfonate-Based Redox Active Ionic Liquid
Source: Materials (Basel). 2021 Jun 12;14(12):3259. doi: 10.3390/ma14123259 (PMC8231554; doi:10.3390/ma14123259)
Supplement: Supplementary file 1 [file materials-14-03259-s001.zip › materials-1230438-supplementary.pdf]

materials

MDPI

Supplementary Information

Synthesis and Characterization of a Novel Hydroquinone Sulfonate-Based Redox Active Ionic Liquid

Farida H. Aidoudi, Alessandro Sinopoli, Muthumeenal Arunachalam, Belabbes Merzougui and Brahim Aïssa *

Qatar Environment and Energy Research Institute (QEERI), Hamad Bin Khalifa University (HBKU), P.O. Box 34110 Doha, Qatar; aidoudifarida@gmail.com (F.H.A.); asinopoli@hbku.edu.qa (A.S.); msundarapandian@hbku.edu.qa (M.A.); belabbes613@gmail.com (B.M.)

* Correspondence: baïssa@hbku.edu.qa or brahim.aissa@mpbc.ca

Electronic Supplementary Information (ESI)

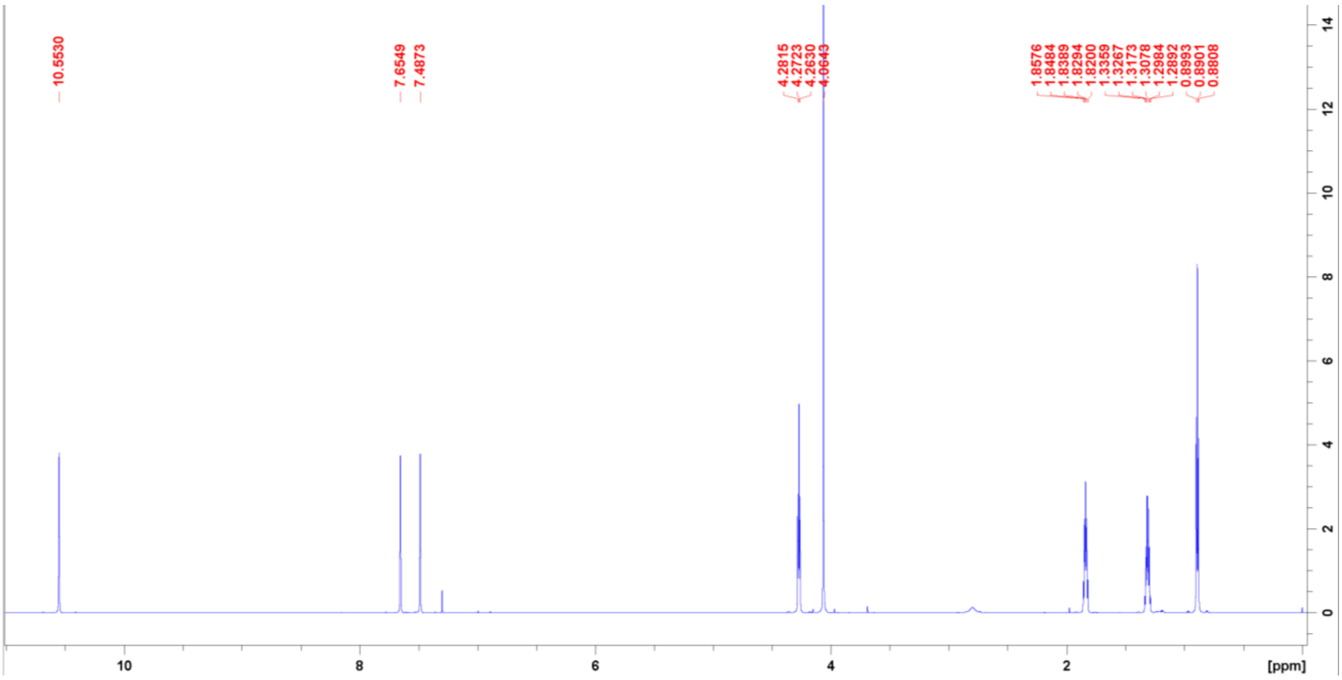

Figure S1. ^{1}H NMR for [BMIM][Cl].

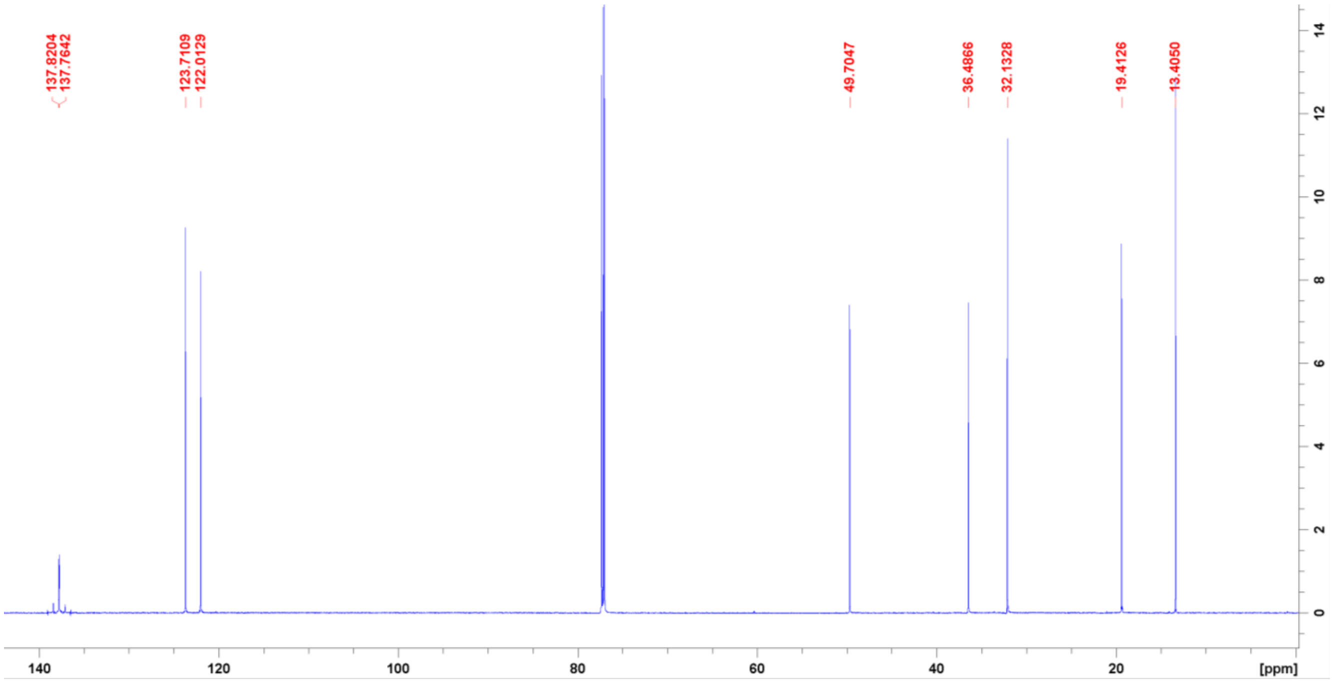

Figure S2. ^{13}C NMR for [BMIM][Cl].

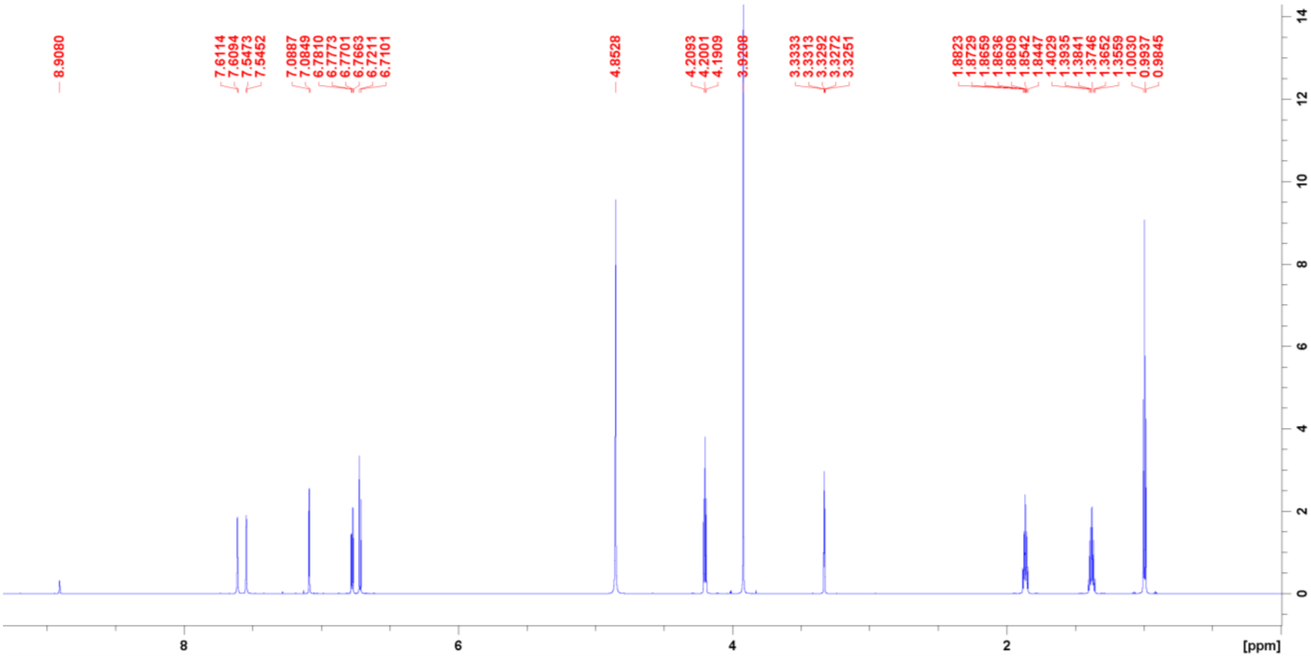

Figure S3. ^{1}H NMR for [BMIM][HQS].

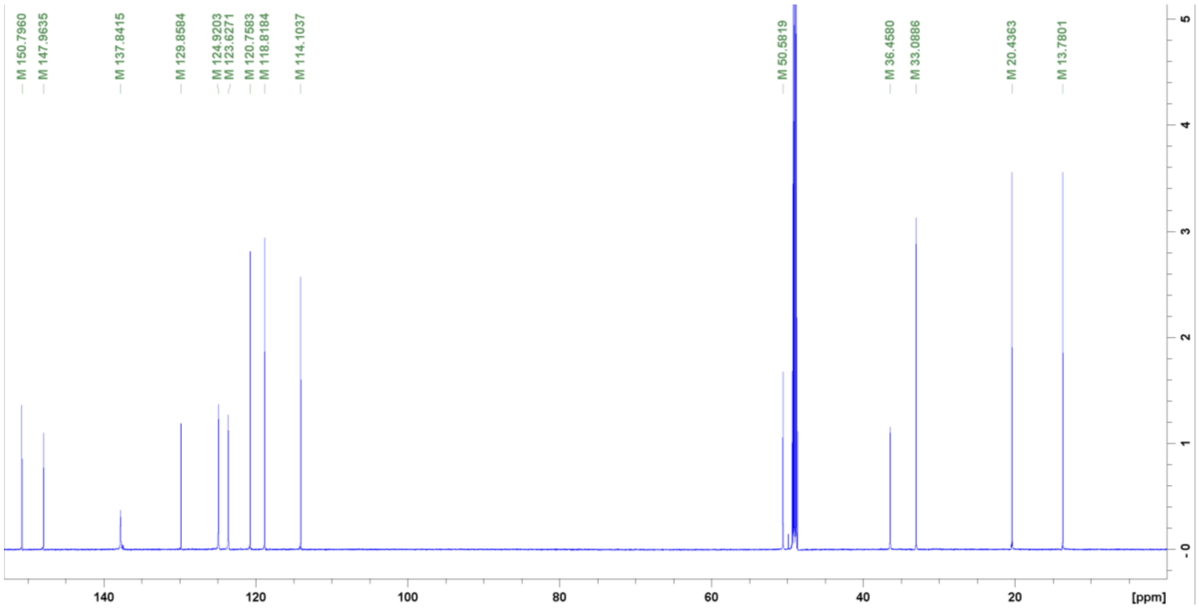

Figure S4. ^{13}C NMR for [BMIM][HQS].

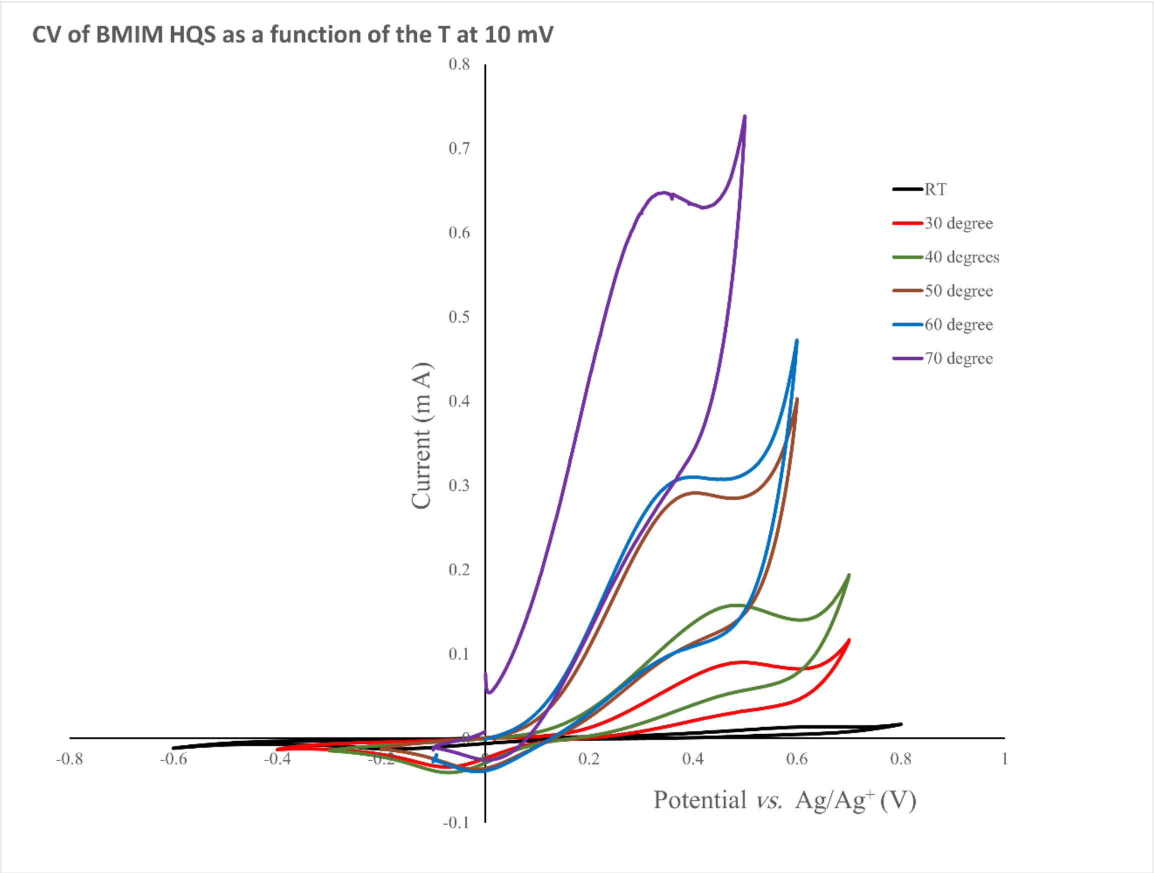

Figure 5. CV of the pure BMIM HQS over the temperature range RT - 70 °C. The scan rate is 10 mV/s, the working electrode is a 300 μm thickness JNTG carbon paper.

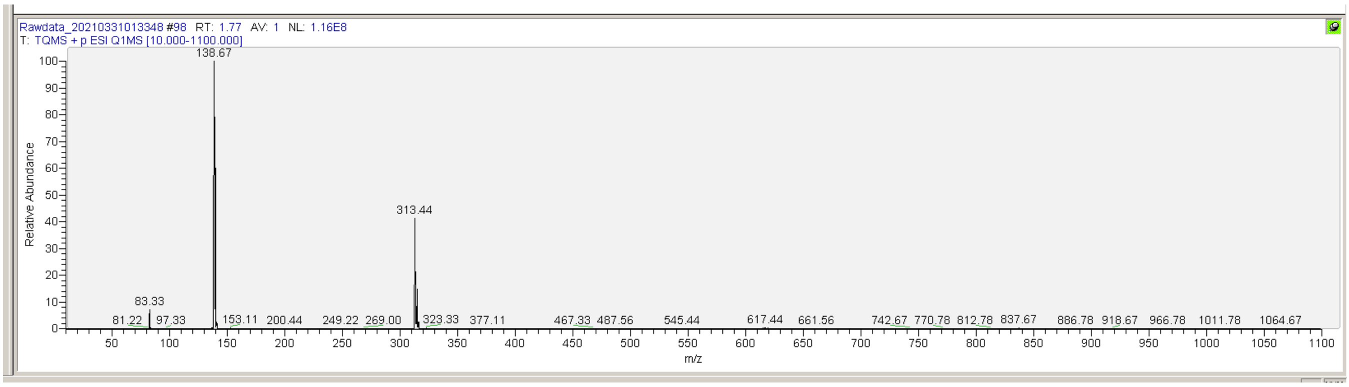

Figure S6. MS spectrum of [BMIM][Cl] in positive mode.

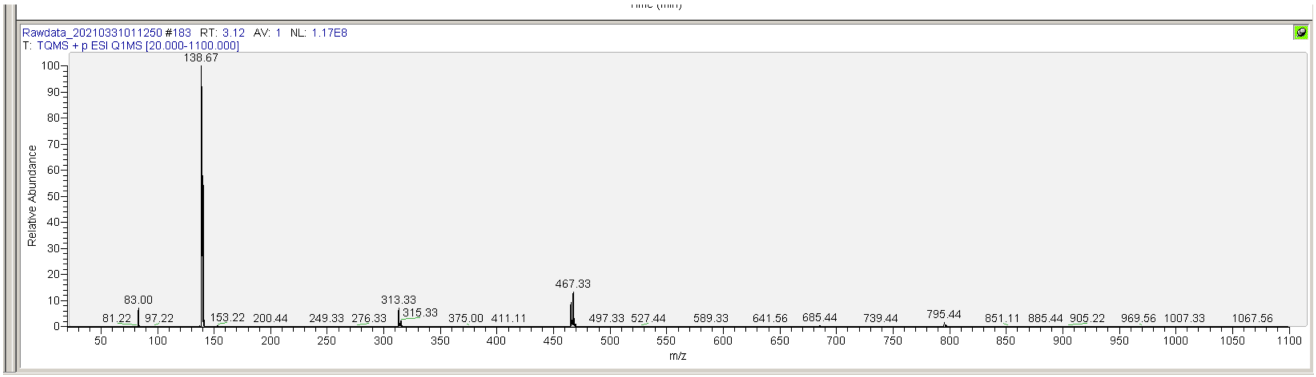

Figure S7. MS spectrum of [BMIM][HQS] in positive mode.

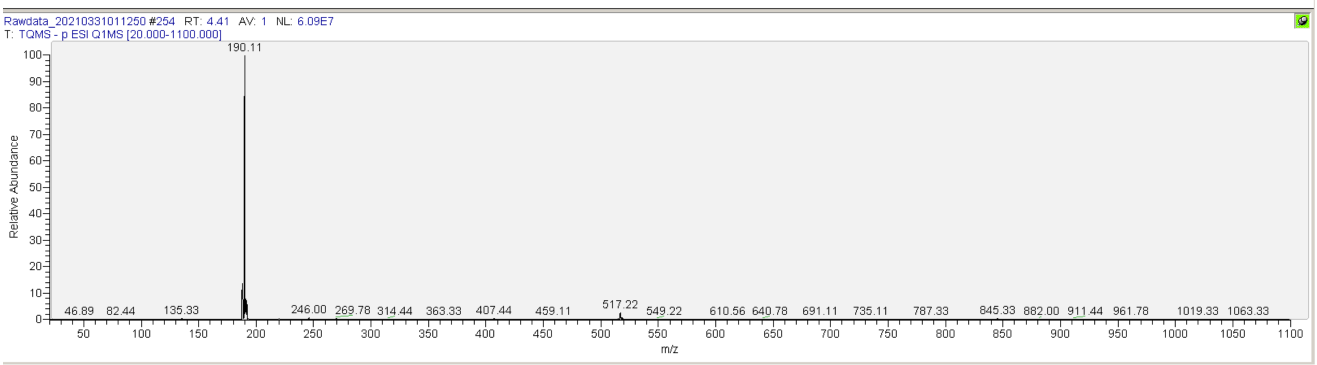

Figure S8. MS spectrum of [BMIM][HQS] in negative mode.
